# Ensemble-based analysis of the pollutant spreading intensity induced by climate change

**DOI:** 10.1038/s41598-019-40451-7

**Published:** 2019-03-07

**Authors:** Tímea Haszpra, Mátyás Herein

**Affiliations:** 1 Institute for Theoretical Physics, Eötvös Loránd University, Budapest, H-1117 Hungary; 2 MTA–ELTE Theoretical Physics Research Group, Budapest, H-1117 Hungary

## Abstract

The intensity of the atmospheric large-scale spreading can be characterized by a measure of chaotic systems, called topological entropy. A pollutant cloud stretches in an exponential manner in time, and in the atmospheric context the topological entropy corresponds to the stretching rate of its length. To explore the plethora of possible climate evolutions, we investigate here pollutant spreading in climate realizations of two climate models to learn what the typical spreading behavior is over a climate change. An overall decrease in the areal mean of the stretching rate is found to be typical in the ensembles of both climate models. This results in larger pollutant concentrations for several geographical regions implying higher environmental risk. A strong correlation is found between the time series of the ensemble mean values of the stretching rate and of the absolute value of the relative vorticity. Here we show that, based on the obtained relationship, the typical intensity of the spreading in an arbitrary climate realization can be estimated by using only the ensemble means of the relative vorticity data of a climate model.

## Introduction

In recent decades there is a growing interest in the potential consequences of climate change. Numerous studies^1–10^ reported that the cyclonic activity and the number of cyclones have changed in the last decades. At the same time the question arises whether quantities related to cyclones—such as the intensity of the spreading of atmospheric pollutant clouds—could also change during a climate change. It has been shown in ref.^11^ that, based on a single set of reanalysis data, a change can be identified from 1979 to 2015 in the areal mean of the intensity of the pollutant spreading, for both the tropics and the extratropics. It has been also reported in that study that a strong correlation is found between the intensity of the spreading and the relative vorticity in the time series of the two quantities.

In general, analyzing historical meteorological reanalysis data, time series analysis is the only tool available to identify trends and to determine correlation coefficients between variables. However, if one intends to perform predictions for the climate system characterized by complex (chaotic), non-stationary dynamics, it has been established that the only way to gain appropriate statistics is evaluating them over an ensemble of parallel climate realizations^12–15^. As a consequence, it might be misleading to study one single time-series, therefore, even the analysis of large-scale spreading needs to be reconsidered. Recent studies, see e.g. ref.^16^, reveal that the analysis of single time-series is not representative, they might exhibit properties being atypical in the whole ensemble. Furthermore, to be able to use any temporal averaging technique one needs stationarity, a condition that does not apply for a changing climate. Therefore, in this paper we turn to the use of ensembles in the sense of the so-called snapshot (or pullback) attractor approach^15,17^.

In practice, this ensemble technique corresponds to studying an ensemble of parallel climate realizations in which the initial conditions of the individual field realizations slightly differ but the dynamics of all realizations are subjected to the same governing equations. Therefore, this approach is similar to ensemble weather forecasts^18^, however, in contrast to them, the time-interval for the climate realizations is long enough for the ensemble members to forget their initial conditions. After a transient time the ensemble correctly characterizes the potential set of typical climate states permitted by the climate dynamics. An introduction to the concept of this ensemble approach can be found in refs^15,17,19–21^, and an experimental implementation of this concept is also available^22^.

In this study the ensemble climate realizations are produced by the Planet Simulator (PlaSim)^23^ intermediate complexity climate model and by one of the state-of-the-art climate models, the Community Earth System Model–Large Ensemble project (CESM–LE)^24,25^. The spreading of the pollutants is simulated by the Real Particle Lagrangian Trajectory (RePLaT) model^26,27^. We emphasize that to our knowledge, this is the first study investigating the change in the intensity of the atmospheric large-scale transport processes in the ensemble approach.

## Setup

In the case of PlaSim an ensemble of 110 climate realizations is used. As the 360 ppm CO_2_ concentration is often used as a reference level in climate studies, representative of the present-day value, and its doubling is a widely used scenario^28,29^ each of the realizations is characterized by a 50-year-long plateau with constant CO_2_ concentration which is followed by a doubling of the CO_2_ concentration from 360 ppm to 720 ppm over 100 years (implying an increase of 6 °C in the ensemble mean of the global mean surface temperature). Because the transient time of the ensemble is found to be about 30 years in earlier studies^14^, the dispersion simulations are carried out from year 30 to year 150.

The results based on the climate realizations of CESM are also analyzed in order to compare with the PlaSim outcomes. In this study 35 members of CESM–LE have been used. For these realizations the ensemble mean of the global mean surface temperature from 1990 to 2080 increases by about 3.5 °C. For the transport simulations the velocity fields of the years 1990–2005, 2026–2035 and 2071–2080 are used in 6 h time resolution (required for the transport calculations). Since the vertical velocity is not available, 2D transport simulations are carried out.

For more details on the PlaSim, CESM–LE and RePLaT models and the setups see the section Methods.

## Characterization of the Spreading—the Stretching Rate

**Figure 1:**
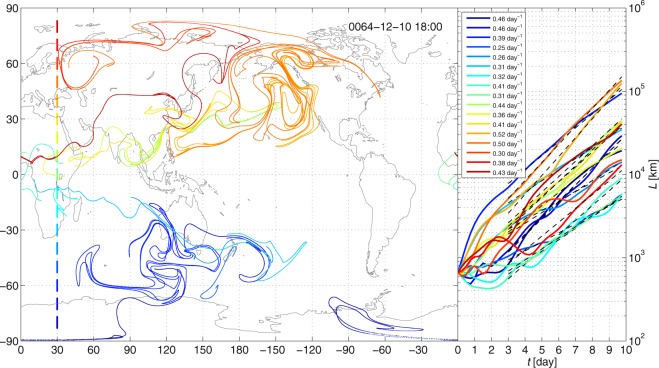
Left: Pollutant patterns. Advection image of 17 pieces of initially 6°-long filaments on the 10th day after the initialization using the meteorological data of the ensemble member *E* = 64 from the PlaSim realizations. Initial conditions: *n*_0_ = 1000 particles initiated on December 1, yr 64 at 00 UTC at 30 °E, 500 hPa from 80 °S to 80 °N in 10° increments (see the thick colored line segments). Right: The length of the filaments in time (solid lines) and the exponential functions fitted from day 3 to 10 (dashed lines). The stretching rate of the filaments are indicated in the legend.

To characterize the intensity of pollutant spreading, the so-called stretching rate is used. Figure 1 illustrates exemplary “pollutant clouds” (ensembles of *n*_0_ particles) initiated as 1-D filaments distributed along a meridian in a single climate realization. It shows that pollutant clouds typically spread in a more and more stretched, filamentary structure in the atmosphere. After a few days, the length *L* of a filament can be approximated by an exponential function over time *t*, that is, *L*(*t*)~exp(*ht*), where the exponent *h* is called the stretching rate. It corresponds to the topological entropy in dynamical systems theory^30,31^. This quantity is a measure of the chaoticity, and, therefore, is closely related to the unpredictability of the spreading and the complexity of the structure of a pollutant cloud. In general, the larger the topological entropy, the faster a pollutant cloud grows and the more foldings and meanders it displays. For more details on the topological entropy, see refs^32,33^, and for the application in atmospheric spreading see refs^11,34^.

In this study, the filaments are tracked for a 10-day-long time period, characteristic to continental and global transport processes. The stretching rate is determined by fitting a linear function to the time evolution of the natural logarithm of the length of a filament from day 3 to 10. (For the technical details, see section Methods). Filaments of initial length of 6° (for PlaSim) and 3° (for CESM–LE) are initialized in the free atmosphere at the level of 500 hPa, so that even the initial length of the line segments exceeds the grid size of the meteorological fields. In order to be able to study the geographical dependence of the stretching rate, in each simulation 12 × 17 filaments are initialized uniformly distributed over the globe from 150 °W to 180 °E and from 80 °S to 80 °N. These simulations are started in every 10 days for June–July–August (JJA) and December–January–February (DJF) for the years 30–150 for each of the 110 PlaSim climate realizations and for the years 1990–2005, 2026–2035 and 2071–2080 for each of the 35 CESM realizations.

## Results

### The change in the stretching rate due to climate change

**Figure 2:**
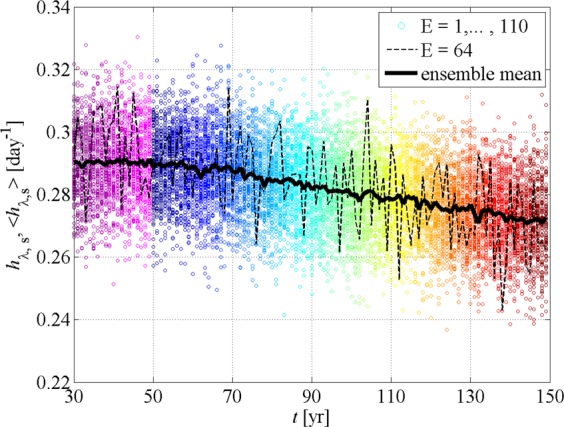
Time-dependence of the stretching rate in different climate realizations. The zonal-seasonal mean *h*_*λ*,*s*_ of the stretching rate *h* and the ensemble mean 〈*h*_*λ*,*s*_〉 of *h*_*λ*,*s*_ for the PlaSim climate realizations at 30 °N in JJA for years 30–49 (before climate change, in the shades of violet) and for years 50–150 (after the outset of the climate change, from blue to red). Colored circles represent the data of the whole ensemble, the ones connected with the dashed line correspond to the member *E* = 64 and the thick solid line indicates the ensemble mean 〈*h*_*λ*,*s*_〉 in Fig. 3a.

Figure 2 illustrates the time series of the zonal-seasonal mean *h*_*λ*,*s*_ of the stretching rates for the latitude of 30 °N for JJA indicating the data of the whole ensemble of PlaSim and also highlighting one particular member for illustrative purposes (*E* = 64). Each color marks one year: the ones in the shades of violet correspond to the years 30–49 of constant CO_2_ concentration and constant surface temperature (see Fig. 5 in Methods), and, therefore, they represent a stationary climate, while colors from blue to red illustrate the years of the doubling of the CO_2_ concentration (years 50–150). When considering only a single member (dashed line) large fluctuations can be seen, and it is hard to decide whether any trend exists for years 30–49. At the same time, taking into account all of the members, it is clear that there is no trend in *h*_*λ*,*s*_ for the years of the stationary climate (violet). For years 50–150, red circles exhibit smaller values than blue ones in general, and also the values along the dashed line become smaller on average, that is, a decrease in the stretching rate can be observed. Studying the time series of the remaining latitudes, we find regions where the existence of a trend cannot be determined by utilizing one single ensemble member, because the fluctuations among the years are also on the order of the assumed trends (see Section 1 in Supplementary information).

**Figure 3:**
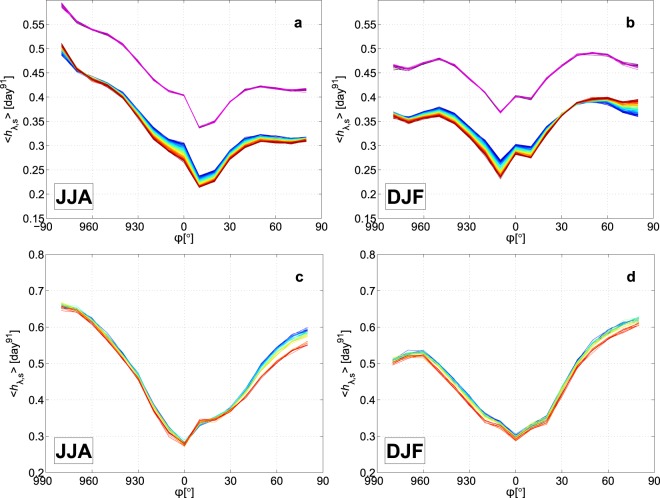
Zonal dependence of the typical stretching rates. The ensemble mean 〈*h*_*λ*,*s*_〉 of the zonal-seasonal mean *h*_*λ*,*s*_ of the stretching rate *h* for the PlaSim (**a**,**b**) and for the CESM climate realizations (**c**,**d**) in JJA (**a**,**c**) and DJF (**b**,**d**) for years 30–49 (in the shades of violet) and for years 50–150 (from blue to red) for PlaSim, and for years 1990–2005, 2026–2035 and 2071–2080 (from blue to red) for CESM realizations. Each line connects the mean of the different latitudes for a given year. The values of the years 30 to 49 (representing the stationary climate) in the panels a,b are shifted by +0.1 day^−1^ for clarity.

Figure 3a,b represent the zonal distributions of the ensemble mean 〈*h*_*λ*,*s*_〉 of the stretching rate *h*_*λ*,*s*_ for the PlaSim realizations. (For the time series of the 30 °N see the thick solid line in Fig. 2). The zonal-seasonal mean stretching rate 〈*h*_*λ*,*s*_〉 displays a typical zonal distribution with smaller values in the tropics and larger values in the extratropics. The largest stretching rates appear in the respective winter season of the hemispheres, especially in the Southern Hemisphere. Based on these curves, the overall changes are much easier to describe: they show that during years 30–49 〈*h*_*λ*,*s*_〉 does not change, and it is also clear from the figure that for years 50–150 a decrease of 0.02–0.05 day^−1^ characterizes almost the whole globe in both seasons except a small band of width of 10° around the Southern Pole in JJA and a band with width of 40° at the mid- and high latitudes of the Northern Hemisphere.

The zonal distributions of the ensemble mean 〈*h*_*λ*,*s*_〉 for the CESM climate realizations are shown in Fig. 3c,d. The shape of the curves is similar for both climate models, which–as a side product–supports the applicability of PlaSim in this context. We note that the reason of the higher 〈*h*_*λ*,*s*_〉 values for CESM is the consequence of the resolution of the utilized velocity fields: the more detail a field contains the more meandering and curly the filament can become, i.e., the results tend to show some dependence on the grid resolution. The 〈*h*_*λ*,*s*_〉 values of the CESM climate realizations show a clear decrease of 0.01–0.05 day^−1^ from 1990 to 2080 for both seasons and for almost the entire globe.

It is worth noting that even the change of ±0.05 day^−1^ in the ensemble mean values leads to 10-day-old filaments with length of 165% and 61% of the filaments without any climate change, and the change can be much larger in individual realizations for individual filaments than the change in the ensemble mean.

### The relationship of the stretching rate and the relative vorticity

The position of the *n*_0_ particles of a filament is determined by the local velocity of the atmospheric flow in the consecutive time instants. Therefore, the length of a filament (computed from the particle positions), and hence the stretching rate *h* of a filament are Lagrangian quantities determined by the velocity values along the filament over the investigated time interval. However, if one intends to estimate the change in the intensity of large-scale spreading based on only the meteorological fields of a climate realization, without carrying out transport simulations and knowing the exact trajectories which the *n*_0_ particles would follow, only Eulerian characteristics from the gridded meteorological data can be calculated. Therefore, the relationship of an Eulerian quantity to the stretching rate is investigated in this study. (As an outlook, in the Supplementary information in Section 2 we also present a brief study in which the relationship of *h* to a meteorological quantity calculated along the filaments is investigated). In a previous research^11^, not using meteorological ensembles, the connection with numerous meteorological quantities was analyzed, and it was found that the correlation is the strongest between the stretching rate and the absolute value of the relative vorticity |*ξ*|. Therefore, in this paper we investigate the relationship of the stretching rate with only this quantity. Note that |*ξ*| is also a quantity directly linked to the velocity differences of the atmospheric flow that cause the stretching of the filaments. A further advantage of relative vorticity is that it is easy and clear to determine based on velocity fields operationally produced by the climate models.

**Figure 4:**
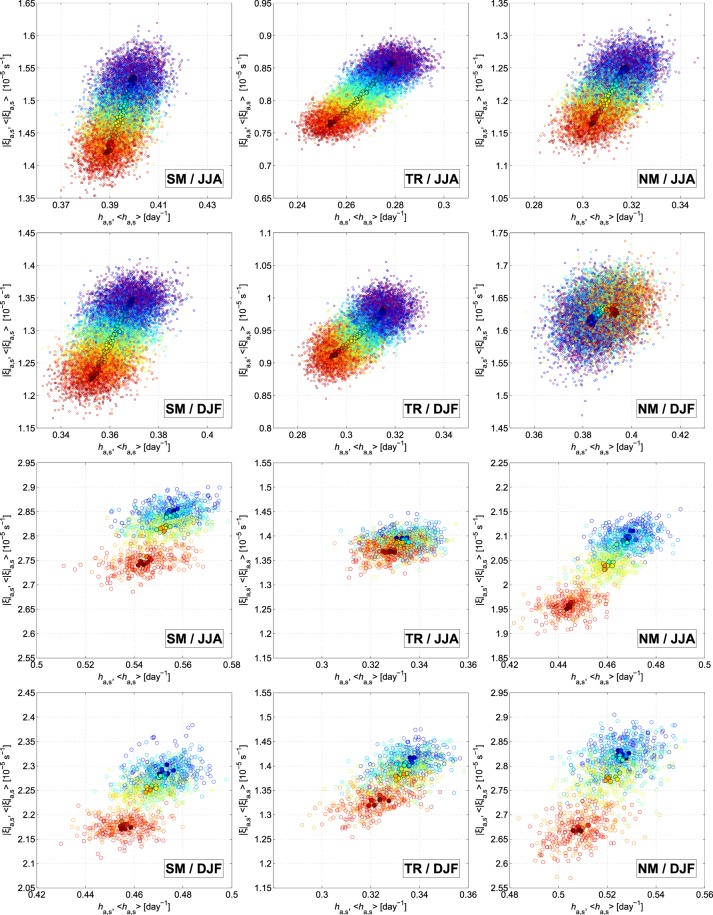
Scatter plots. Diagrams for the areal-seasonal mean stretching rate *h*_*a*,*s*_ and its ensemble mean 〈*h*_*a*,*s*_〉 versus the areal-seasonal mean of the absolute value of the relative vorticity |*ξ*|_*a,s*_ of the level 500 hPa and its ensemble mean 〈|*ξ*|_*a*,*s*_〉 for JJA and DJF and for SM, TR and NM for the PlaSim (upper 6 panels) and CESM (lower 6 panels) climate realizations. Years are colored as in Figs 2 and 3. Empty circles correspond to the individual ensemble members and filled circles with black edge correspond to the ensemble means.

We found that a particularly illuminating way of expressing this relationship is to plot the areal-seasonal mean *h*_*a*,*s*_ vs. the areal-seasonal mean of the absolute value of the relative vorticity |*ξ*|_*a*,*s*_ for each of the PlaSim and CESM climate realizations, respectively. In Fig. 4 these data are plotted as empty circles. The ensemble mean values of these quantities (filled circles) for each year are also displayed in the panels. Circles are colored from blue to red according to the year they represent (see figure caption for the details). The globe is divided into three belts (SM/NM: mid- and high latitudes of Southern/Northern Hemisphere, and TR: tropical belt) for which we assume that their areal-seasonal mean |*ξ*|_*a*,*s*_ principally influences the stretching of the filaments initialized there. For the CESM simulations the three belts are symmetric and range as 90 °S–30 °S (SM), 30 °S–30 °N (TR) and 30 °N–90 °N (NM), respectively. For the PlaSim climate realizations, in order not to smooth out the values of the latitudes of the Northern Hemisphere with the definite increasing and the ones with decreasing trends, we choose the boundaries as 90 °S–25 °S (SM), 25 °S–40 °N (TR) and 40 °N–90 °N (NM), respectively.

The upper 6 panels of Fig. 4 illustrate that during the time-period of the stationary climate (violet) no trend can be seen either for the stretching rate (as stated earlier, based on Fig. 3a,b) or for the absolute value of the relative vorticity of the 500 hPa level. During the time-period of the CO_2_ ramp (blue to red) in all panels (excluding NM/DJF), both *h*_*a*,*s*_ and |*ξ*|_*a*,*s*_ decrease in parallel. The ensemble mean pairs of 〈*h*_*a*,*s*_〉 and 〈|*ξ*|_*a*,*s*_〉 reveal the relation much more clearly as they are located along straight lines with a decrease of 0.01–0.03 day^−1^ and [0.1–0.15] × 10^−5^ s^−1^, respectively. Therefore, it makes sense to fit regression lines to the ensemble mean data. The slope of the fits and the correlation coefficients calculated for (〈*h*_*a*,*s*_〉, 〈|*ξ*|_*a*,*s*_〉) for the 300, 500 and 800 hPa levels are presented in Table 1 and show a strong correlation between the stretching rate and relative vorticity with correlation coefficients beyond 0.93. Although the “outlier” panel of NM/DJF in Fig. 4 with 〈|*ξ*|_*a*,*s*_〉 calculated from the relative vorticity of the 500 hPa level does not trace out a straight line and therefore its correlation coefficient is much lower than the others ones’, Table 1 shows that even for this case a very well-fitting linear relation between 〈*h*_*a*,*s*_〉 and 〈|*ξ*|_*a*,*s*_〉 can be found utilizing the relative vorticity of other pressure levels.

**Table 1:** The slope *a* of the linear regression and the correlation coefficient *R*_*t*_ between the ensemble mean 〈*h*_*a*,*s*_〉 and 〈|*ξ*|_*a*,*s*_〉 for different pressure levels *p* for JJA and DJF and for SM, TR and NM calculated from year 50 to 150 in the PlaSim simulations.

*p* [hPa]	300	500	800	300	500	800	300	500	800
*a* [10^−5^ s^−1^ day]	SM			TR			NM		
JJA	5.186	10.223	4.418	1.858	3.756	2.245	1.354	6.123	4.207
DJF	4.956	7.379	5.560	1.966	3.268	4.151	4.994	1.465	13.309
***R***_***t***_									
JJA	0.981	0.981	0.949	0.996	0.996	0.995	0.938	0.988	0.981
DJF	0.991	0.990	0.987	0.995	0.993	0.988	0.961	0.689	0.963

In the case of the CESM climate realizations, even if we do not have continuous data for the time-period of 1990–2080, the lower 6 panels of Fig. 4 strengthen our assumption that the stretching rate and the absolute value of the relative vorticity change in parallel in time. Similarly to most of the geographical regions in the PlaSim, 〈*h*_*a*,*s*_〉, *h*_*a*,*s*_ and 〈|*ξ*|_*a*,*s*_〉, |*ξ*|_*a*,*s*_ decrease with the increasing global mean surface temperature. In general, the decrease falls between 0.01 and 0.02 day^−1^, and 0.05 × 10^−5^ and 0.1 × 10^−5^ s^−1^, respectively. A slightly greater variability in the ensemble mean values of subsequent years can be seen in the lower 6 panels than in the upper 6 panels of Fig. 4 which is due to the much fewer members of the ensemble for the CESM simulations. Based on the results of the PlaSim simulations assuming a linear relation between the stretching rate and the relative vorticity, the slopes of the linear fits to the ensemble mean data and the corresponding correlation coefficients are presented in Table 2. The slopes are similar in values to those obtained from the PlaSim simulations with a mean of approximately [6–7] × 10^−5^ s^−1^ day, and, furthermore, all of them are on the order of magnitude of the slope found for the relation of the stretching rate and the relative vorticity in Section 2 of the Supplementary information.

**Table 2:** The slope *a* of the linear regression and the correlation coefficient *R*_*t*_ between the ensemble mean 〈*h*_*a*,*s*_〉 and 〈|*ξ*|_*a*,*s*_〉 for JJA and DJF and for SM, TR and NM calculated from year 1990 to 2080 in the CESM simulations.

*a* [10^−5^ s^−1^ day]	SM	TR	NM
JJA	8.293	3.098	5.688
DJF	6.388	5.549	8.261
***R***_***t***_			
JJA	0.960	0.831	0.991
DJF	0.978	0.949	0.971

## Discussion

We found both for the PlaSim and for the CESM climate realizations that the ensemble mean of the zonal-seasonal mean stretching rate decreases for almost all of the latitudes with the increase of the global mean surface temperature. It implies that the proportion of the mean length of 10-day-old filaments of the later years (*L*_later_) and that of the former years (*L*_former_) decreases to 60%. In individual realizations, this change might be somewhat larger and the ratio might fall below 40%. Our results imply that the intensity of the spreading and, therefore, the typical extension of a polluted region from a pollution event decreases almost everywhere on the globe. We note that the decrease of the spreading might cause larger pollutant concentration for several regions, resulting in higher environmental risk. For completeness, we mention that in some regions, typically near the poles PlaSim shows an opposite trend, where *L*_later_/*L*_former_ even exceeds to 270%.

The results obtained by using the meteorological fields of both models agree in the fact that the ensemble mean of the areal-seasonal mean of the stretching rate and that of the absolute value of the relative vorticity change in parallel, and there is a clear linear dependence between them with an average slope of [6–7] × 10^−5^ s^−1^ day and correlation coefficients beyond 0.93 for the PlaSim and 0.83 for the CESM ensembles, respectively. This relationship may help estimate the changes in the intensity of spreading for arbitrary ensembles utilizing only meteorological variables (i.e. relative vorticity or velocity data) operationally computed and stored by the climate models, without carrying out numerous computationally demanding dispersion simulations. It is interesting to note that a similar relation between some kind of stretching, i.e., the relative dispersion of particle pairs (the analogs of Lyapunov exponents in the language of dynamical system theory describing the local, short term stretching) and enstrophy was recognized even in the early 70’s^35^. However, that investigation is valid only for homogeneous two-dimensional turbulence, which is not our case.

To summarize, in this study we investigated the large-scale spreading in an ensemble approach. Our results reveal that in order to gain the typical features of spreading under different climate conditions, it is unavoidable to use the ensemble framework proposed here. For an improvement, higher resolution meteorological fields and ensembles of climate realizations of at least hundreds of members would be needed.

## Methods

### Models and Data

#### The PlaSim climate model

PlaSim is an intermediate complexity climate model with scalable and modular structure, therefore, it is an ideal tool to study the large-scale features of the climate dynamics, see, e.g. refs^14,16^. PlaSim is based on the moist primitive equations and contains parametrization for the unresolved processes. For the details see ref.^23^. In this study for an ensemble of 110 climate realizations the default PlaSim setup is used at T21 horizontal resolution (corresponding to about 5.6° × 5.6° on a Gaussian grid with 64 × 32 grid points) with 10 sigma levels and 10 minutes of time resolution.

**Figure 5:**
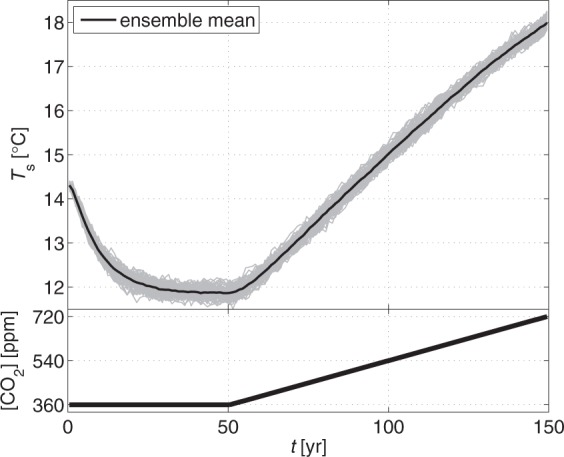
The prescribed CO_2_ concentration (bottom) and the global mean surface temperature *T*_*s*_ (top) in the PlaSim simulations. Individual ensemble members are marked by gray, the ensemble mean is marked by a thick black curve.

To study the change during a transition between two significantly different climate states (which differ much more than the climate of the 1980s and 2010s in the previous study of ref.^11^) we prescribe the CO_2_ concentration scenario as a 50-year-long plateau with constant CO_2_ concentration which is followed by a doubling of the CO_2_ concentration from 360 ppm to 720 ppm over 100 years. For illustration, see the bottom panel of Fig. 5. This implies an increase of about 6 °C in the ensemble mean of the global mean surface temperature in the simulations (see the top panel of Fig. 5). The transient time is clearly visible as all of the realizations converge to a stationary state approximately after year 30. This time-independent state lasts up to year 50. The ensemble standard deviation of the global mean surface temperature, that characterizes the internal variability of the climate, is 0.09–0.13 °C.

#### The CESM–LE model

The CESM is a state-of-the-art CMIP5 (Coupled Model Intercomparison Project Phase 5) climate model. The CESM community designed the CESM Large Ensemble (CESM–LE) with the explicit goal of enabling assessment of climate change in the presence of internal climate variability. All CESM–LE realizations use a single model version (CESM with the Community Atmosphere Model, version 5) at a resolution of 192 × 288 (1.25° × 0.94°) in latitudinal and longitudinal directions, respectively, and at 30 vertical levels. The core simulations “replay” approximately two centuries, years 1920–2100, under an external forcing that is historical^36^ up to 2005 and follows the representative concentration pathway 8.5 (RCP8.5)^37^ afterward. The ensemble members have small differences in the initial conditions. In this study the first 35 members of CESM–LE have been used because the remaining 5 members show a small systematic difference from the others. For the utilized realizations the ensemble mean of the global mean surface temperature from 1990 to 2080 increases by about 3.5 °C. For the details of the CESM–LE project see ref.^25^.

#### The RePLaT dispersion model

For the simulation of the spreading of the pollutants in the atmosphere the RePLaT model is used. RePLaT is a Lagrangian trajectory model that tracks individual spherical particles with fixed, realistic radius and density. The velocity of a particle is given by the Newtonian equation of motion of the particle, and in the vertical direction deposition is also taken into account. RePLaT can reckon with the effect of turbulent diffusion on the particles as a stochastic term in the equations of motion, and it can simulate the scavenging of particles by precipitation as a random process that results in a particle being captured by a raindrop with a probability depending on the precipitation intensity and the collision efficiency of raindrops and aerosol particles.

In this paper, the spreading of ideal tracers, corresponding to inert gases, are investigated. For such particles, the velocity of a particle coincides with the velocity of the air at the location of the particle at any time instant. We proved in ref.^34^ that the effect of turbulent diffusion is negligible on the stretching process for large-scale atmospheric dispersion events, therefore, we also neglect it in this study in the calculation of the transport of the pollutants. As the motion of ideal tracers is studied, if in the 3D simulations (PlaSim) in those exceptional cases when a particle encounters with the surface, it bounces back to the atmosphere by a perfectly elastic collision. RePLaT determines the particle trajectories using Euler’s method, where the time step is the same Δ*t* = 45 min as in refs^11,34^, as it proved to be sufficiently small for free atmospheric transport simulations.

### The computation of the stretching rate

The filaments are initiated as meridional line segments and consist of *n*_0_ = 10^3^ uniformly distributed particles. Each filament is tracked for 10 days—a time period that is characteristic to continental and global transport processes—during which if the distance of two neighboring particles becomes larger than 10 km, a new particle is inserted between them. The length of a filament is the sum of the distances of its neighboring particle pairs, and these distances are calculated similarly as in ref.^11^, that is, along great circles neglecting the vertical stretching which proved to be 10^−2^ to 10^−3^ times smaller than the horizontal one^34^:

1 $$L(t)=\sum _{i=1}^{{n}_{0}-1}\,|{{\bf{r}}}_{{\rm{p}},i}(t)-{{\bf{r}}}_{{\rm{p}},i+1}(t)|,$$

where **r**_p,*i*_ is the horizontal position of the *i*th particle and

2 $$\begin{array}{rcl}|{{\bf{r}}}_{{\rm{p}},i}(t)-{{\bf{r}}}_{{\rm{p}},i+1}(t)| & = & \arccos \,[\sin \,{\phi }_{{\rm{p}},i}\,\sin \,{\phi }_{{\rm{p}},i+1}+\,\cos \,{\phi }_{{\rm{p}},i}\,\cos \,{\phi }_{{\rm{p}},i+1}\,\cos \,({\lambda }_{{\rm{p}},i}-{\lambda }_{{\rm{p}},i+1})]\\  &  & \times \,\frac{180}{\pi }\times 111.1,\end{array}$$

where *λ*_p,*i*_ and *φ*_p,*i*_ are the longitudinal and latitudinal coordinate of the *i*th particle, respectively, and $\frac{180}{\pi }\times 111.1$ converts the unit from radian to kilometer using the fact that the spherical distance of 1° along a great circle corresponds to a length of 111.1 km along the surface.

## Supplementary information

Supplementary information

## Data Availability

The datasets generated during and analysed during the current study are available from the corresponding author on reasonable request.
