# Supplementary material for: Ensemble-based analysis of the pollutant spreading intensity induced by climate change
Source: Sci Rep. 2019 Mar 7;9:3896. doi: 10.1038/s41598-019-40451-7 (PMC6405940; doi:10.1038/s41598-019-40451-7)
Supplement: Supplementary file 1 — Supplementary information [file 41598_2019_40451_MOESM1_ESM.pdf]

Supplementary information for “Ensemble-based analysis of the pollutant spreading intensity induced by climate change”

Tímea Haszpra* and Mátyás Herein†

1 Time-dependence of the stretching rate

Fig. 1 illustrates that in some regions the existence of a trend cannot be determined by utilizing one single ensemble member since the fluctuations among the years are also on the order of the assumed trends. As an example, for the years 50–150 the ensemble mean follows a clear trend with a slope of 6.85×10^{-5} day^{-1} yr^{-1} significant at a 95% confidence level (p-value $= 0.000$, $R = 0.754$), while the time series of a single member ($E = 64$) does not show any significant trends (p-value $= 0.228$, $R = 0.128$).

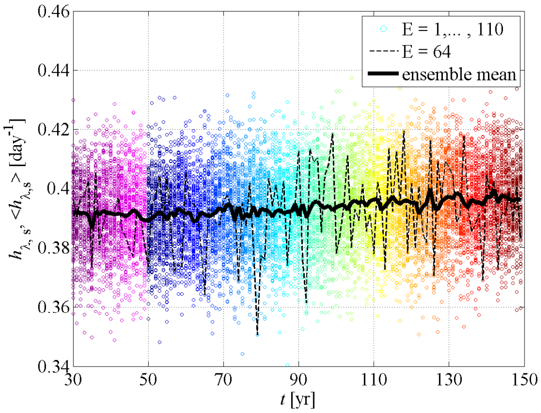

Figure 1: Time-dependence of the stretching rate in different climate realizations. The zonal-seasonal mean $h_{\lambda,s}$ of the stretching rate h and the ensemble mean $\langle h_{\lambda,s}\rangle$ of $h_{\lambda,s}$ for the PlaSim climate realizations at $50°$ N in DJF for years 30–49 (before climate change, in the shades of violet) and for years 50–150 (after the outset of the climate change, from blue to red). Colored circles represent the data of the whole ensemble, the ones connected with the dashed line correspond to the member $E = 64$ and the thick solid line indicates the ensemble mean $\langle h_{\lambda,s}\rangle$ in Fig. 3.b shown in the main text.

2 Relationship of the stretching rate to the relative vorticity in a Lagrangian view

Fig. 2 illustrates stretching rate h and relative vorticity $|\xi|_{L,t}$ pairs where $|\xi|_{L,t}$ is determined by averaging the absolute value of the relative vorticity along the filament and over time, for filaments initialized in the $E = 1$ realization of CESM in yr 1 in every 10 days in JJA in the global distribution described in the main text. The plot indicates a relationship between h and $|\xi|_{L,t}$ with a correlation coefficient of $R = 0.563$. The slope of the linear regression is 2.3×10^{-5} s^{-1} day.

*hatimi@caesar.elte.hu, Institute for Theoretical Physics, Eötvös Loránd University and MTA–ELTE Theoretical Physics Research Group, Eötvös Loránd University, Budapest, H-1117, Hungary

†hereinm@gmail.com, Institute for Theoretical Physics, Eötvös Loránd University and MTA–ELTE Theoretical Physics Research Group, Eötvös Loránd University, Budapest, H-1117, Hungary

This connection proves to be stronger than the ones we also tested, that is, the maximum value in time of $|\xi|$ averaged in each time step along the filament ($R = 0.485$) and the time-mean value of the maximum of $|\xi|$ along the filament in each time step ($R = 0.455$). Based on these results and the fact that a single value of a seasonal or areal maximum of $|\xi|$ would only characterize a single time instant or a single location that do not influence the majority of the evolution of a filament, we choose to study the relationship between the areal and time-mean values of $|\xi|$ and h.

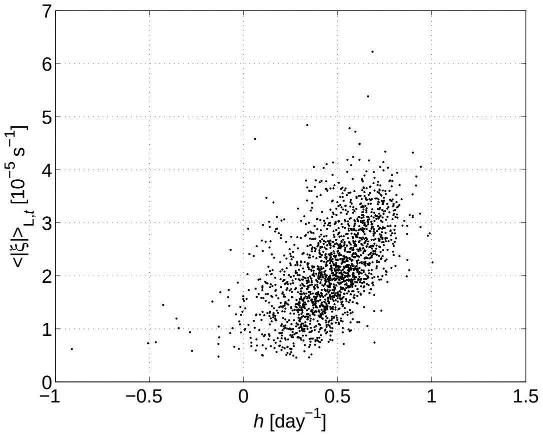

Figure 2: Scatter plot diagram for the stretching rate h of individual filaments versus the absolute value of the relative vorticity $|\xi|_{L,t}$ averaged along the filament and over time. $9 \times 12 \times 17$ filaments are used for the plot that are initialized in the $E = 1$ realization of CESM in yr 1 in every 10 days in JJA in the global distribution described in the main text.
